# The influence of income and testosterone on the validity of facial width-to-height ratio as a biomarker for dominance

**DOI:** 10.1371/journal.pone.0207333

**Published:** 2018-11-09

**Authors:** Emilou Noser, Jessica Schoch, Ulrike Ehlert

**Affiliations:** 1 Clinical Psychology and Psychotherapy, Institute of Psychology, University of Zurich, Zurich, Switzerland; 2 University Research Priority Program (URPP) Dynamics of Healthy Aging, University of Zurich, Zurich, Switzerland; Macquarie University, AUSTRALIA

## Abstract

Research has indicated that men's facial width-to-height ratio (fWHR) is part of an evolved system of social dominance, aggression, and power. fWHR has been linked to antisocial behavior, measured by self-reported aggression, but recent studies have failed to replicate this finding. To overcome these inconsistencies, influencing factors such as social status have to be taken into account in order to explain the relationship between fWHR and aggression. In particular, income has been shown to be an important influencing factor in this relationship. Furthermore, previous findings suggested that testosterone is linked to fWHR and might be associated with fWHR and dominance-related outcomes. Therefore, this study examined the influence of both social status defined by income and salivary testosterone on the association between fWHR and self-reported dominance-related behavioral traits. In particular, links between fWHR and self-report measures of aggression and the Dark Triad (encompassing psychopathy, Machiavellianism, and narcissism) were investigated in *N* = 109 men aged 40 to 75 years. fWHR was significantly associated with physical aggression and two of the Dark Triad traits (psychopathy and Machiavellianism) in men reporting low income. The relationship between fWHR and narcissism was moderated by testosterone. The findings highlight the importance of considering social status and neuroendocrine parameters such as testosterone when examining associations between fWHR and complex psychological traits and behaviors.

## Introduction

A rapidly growing amount of research has established associations between men’s facial width-to-height ratio (fWHR) [1] and a broad range of personality traits and behaviors. In particular, studies have indicated that higher fWHRs are associated with socially undesirable personality characteristics and dominance-related behaviors, including being less trustworthy [2], more self-centered and deceptive [3] [4], more psychopathic [5], and more aggressive [6].

Carré and McCormick [7] demonstrated a positive association between fWHR and aggressive behavior in professional hockey players. The authors suggested that fWHR may be an honest signal of propensity for aggression, which has been supported by recent research: fWHR was related to self-reported aggression as measured by the Buss-Perry Aggression Scale [8] [9] and correlated strongly with judgments of aggression made by observers [10]. However, some studies have failed to replicate the link between fWHR and aggression. Deaner, Goetz, Shattuck, and Schnotala [11] demonstrated that body weight, but not fWHR, predicts aggressive behavior in professional hockey players. Oezener [12] found no association between fWHR and self-reported aggression as measured by the Buss-Perry Aggression Scale. The reasons for these inconsistencies are still unclear. One possible reason lies in the fact that most of the studies did not control important influencing factors. Muñoz-Reyes, Gil-Burmann, and Turiegano [13] stated that fWHR should only be used after controlling for body mass index (BMI). More importantly, although social status is considered as an important factor influencing the relationship between fWHR and aggression [14], none of the aforementioned studies considered social status in their analyses of the relationship between fWHR and aggression.

Besides aggression, fWHR may be related to antisocial personality traits such as psychopathy, Machiavellianism, and narcissism, also known as the Dark Triad. These three disagreeable and aversive constellations of personality traits share a common core of significant harm to or exploitation of others [15] [16]. Even at subclinical levels, psychopathy (callous, impulsive, and predatory behaviors), narcissism (excessive ego and selfish behavior), and Machiavellianism (calculated social manipulation) are associated with significant social, emotional, and legal harm [16]. Besides the harm they cause to others, these personality traits may yield significant immediate and evolutionary benefits [15]. Each of the three Dark Triad personality traits is characterized by ruthless self-advancement [17], which may exploit the evolved cooperative behaviors of most people, while eliminating the evolved need to reciprocate [18].

So far, the relationship between fWHR and the Dark Triad has not been examined. However, Anderl et al. [5] found a positive correlation between fWHR and psychopathy, measured using the Psychopathic Inventory-Revised [19]. Since fWHR has been shown to be associated with other socially undesirable personality traits such as being untrustworthy or deceptive [3] [4] [2], we assume a positive association between fWHR and the Dark Triad. Previous research has indicated that both fWHR [20] and the Dark Triad [21] are part of an evolved system of social dominance and power. With regard to the Dark Triad, this evolved system serves as a short-term and exploitative social strategy that emphasizes personal gains at the expense of cooperation [21]. Similar to aggression, the Dark Triad personality traits can therefore be seen as risk-taking strategies to fulfill the needs of men with high fWHRs for power and social dominance.

Social status is considered as one important factor influencing the relationship between fWHR and dominance-related traits [14]. High social status is defined in this regard as having greater personal resources, such as higher income [22]. Although income and education are both facets of social status, they constitute distinct bases of hierarchical differentiation [23]. Income, but not educational level, was found to predict unethical behavior [24]. Moreover, income is related to control over valued resources and is therefore more likely to be intrinsically linked to dominance and power. Vohs, Mead, and Goode [25] found that exposure to money can lead to similar consequences to power, such as an increase in self-focus. In addition, a lack of power increases people's need for money [26].

Based on the hypothesis that fWHR is part of an evolved cueing system of social dominance and power [20], men with high fWHRs should display a greater need for power and be more willing to take risks to achieve resources such as money. A high income might increase feelings of power and dominance and may therefore act as a source of power in men with high fWHRs. By contrast, men with high fWHRs but low income might be faced with a discrepancy between their needs for power and social dominance and their resources. Due to their elevated need for social dominance and power, compared to men with low fWHRs, men with high fWHRs should be more likely to engage in dominance-related behaviors in situations of high need such as low income. Indeed, there is initial evidence highlighting the influence of income on the relationship between fWHR and dominance. Goetz et al. [14] showed that income moderated the relationship between fWHR and aggressive behavior and confirmed that fWHR is a robust predictor of aggression, but only in the context of a low income. The authors attributed this finding to the distribution of the costs and benefits of aggression across social strata: For high-status men, the costs of aggression may outweigh the potential benefits; thus, in comparison to low-status men, those with a high status may have much more to lose from aggressive behavior and relatively little to gain. The role of social status as a moderator of the relationship between fWHR and social dominance has also been documented in non-human primates [27]. Carré [28] showed that fWHR was only related to dominance-related behaviors among low-status monkeys. Likewise, a recent human study demonstrated moderating effects of social status on the relationship between fWHR and risk-taking in men [29]. Overall, these findings highlight the importance of taking into account social status when examining the link between fWHR and dominance-related traits and behaviors.

As one of the most prominent male hormonal parameters, testosterone has been proposed as a common underlying factor linking fWHR to dominance-related traits and behaviors [30, 31, 32, 33]. Testosterone affects both the craniofacial growth and the expression of behavioral traits as part of sexual differentiation in adolescence, and might therefore be responsible for the link between fWHR and dominance-related traits. Indeed, baseline and reactive testosterone levels in men were positively linked to fWHR [30], and baseline testosterone was positively related to self-reported dominance [34]. However, a recent analysis did not reveal a significant positive relationship between fWHR and baseline testosterone [35]. These inconsistent findings might be attributable to the failure to include relevant co-variables such as BMI. An alternative explanation might be that fWHR maps more onto the secretion of testosterone during adolescence [33]. The influence of adult testosterone on the relationship between fWHR and social dominance is still unclear. Based on the assumption that both fWHR and adult testosterone each exerts its own specific effect on social dominance in adulthood, we hypothesize that adult testosterone and fWHR are not directly related but that their effects interact. To date, no study has investigated the potential moderating role of testosterone in the relationship between fWHR and social dominance.

Taken together, a high fWHR seems to be a relevant biomarker for dominance-related outcomes. However, previous findings indicated that fWHR is positively associated with aggression only at a low income. It has not yet been examined whether income influences the relationship between fWHR and self-reports of dominance-related traits such as self-reported aggression and the Dark Triad. Finally, the role of testosterone in the link between fWHR and dominance-related traits remains unclear.

### Present research

The primary aim of the present study was to explore the relationship between fWHR and self-reported dominance-related traits: a broad range of aggression (physical, verbal, anger, hostility) and the Dark Triad. This is the first study to investigate whether the association between fWHR and these dominance-related traits depends on social status as defined by income. We assumed a positive relationship between fWHR and the dominance-related traits only at a low income. Furthermore, the study is the first to examine the potential interaction between the effects of both fWHR and adult testosterone on social dominance. We expected testosterone to be correlated with fWHR and to be an important influencing factor in the relationship between fWHR and the dominance-related traits.

## Methods

The data for the present study were collected in the framework of a cross-sectional project entitled Men Stress 40+. In the following, only procedures and measures used for the present study are described. For more detailed information about the project, see Noser, Fischer, Ruppen, and Ehlert [36].

### Participants

A total of 123 men provided psychometric and biological data. The sample size was determined with G-Power calculation [37]. For multiple linear regression, an effect size of *f*^**2**^ = .15, alpha level of .05, and number of predictors = 3, a power of .95 would be achieved with a sample size of *N* = 74. The final sample size of *N* = 123 provided a well-powered (.95) analysis. Participants were recruited through local online platforms, newspaper announcements, and flyers. In addition to male sex and age between 40 and 75 years; inclusion criteria for study participation were no self-reported mental disorder or physical disease, no consumption of psychotropic substances during the past two months, no psychotherapeutic intervention in the past six months, and no more than two standard units of alcoholic beverages per day.

In order to only include participants with a current working income, retired men were excluded from data analyses (*n* = 13). Additionally, one subject did not provide consent for the facial image and was therefore excluded. The final sample consisted of *N* = 109 men currently residing in Switzerland or Germany. Participants had an average age of 50.92 years (*SD* = 6.71). Forty-four percent (*n* = 48) of the sample had attained tertiary education, including universities as well as trade schools and college. The annual gross income ranged from 18,000 to 700,000 Swiss francs, with a mean of 137,972.94 (*SD* = 88,717.26). The participants had a mean body mass index of 25.77 (*SD* = 3.83). With respect to medication intake, 80% (*n* = 87) of the sample did not take any medication; the most frequently taken medications in the remaining participants were antihypertensive drugs (*n* = 15) and medications to treat high cholesterol (*n* = 6). All participants provided written informed consent prior to study participation. The Ethics Committee of the Canton of Zurich approved the study protocol before data collection.

### Measures

#### Facial width-to-height ratio

Prior to measurement, all pictures were horizontally aligned using Fotor, a software program for editing images (http://www.fotor.com). fWHR was measured by calculating the ratio of bizygomatic width (maximum horizontal distance from the left facial boundary to the right facial boundary) to upper-face height (vertical distance from the highest point of the upper lip to the highest point of the eyelids) from pictures [2, 30]. Two trained raters measured fWHR using the National Institutes of Health open-access ImageJ software (http://rsbweb.nih.gov/ij/). Both raters were blind to the research questions. The measures were highly consistent (*α* = .99) and were thus averaged into one index of fWHR.

#### Dark triad

Participants rated themselves on psychopathy (e.g. I tend to lack remorse; *α* = .56), Machiavellianism (e.g. I have used deceit or lied to get my way; *α* = .70), and narcissism (e.g. I tend to want others to admire me; *α* = .76) using the Dirty Dozen scale [38] in its German version [39]. The Dirty Dozen is composed of 12 items rated on a nine-point Likert scale (1 = totally disagree, 9 = totally agree).

#### Aggression

Participants rated themselves on physical aggression (e.g. I get into fights a little more than the average person; *α* = .60), verbal aggression (e.g. I often find myself disagreeing with people; *α* = .62), anger (e.g. I flare up quickly but get over it quickly, *α* = .81), and hostility (e.g. I wonder why sometimes I feel so bitter about things, *α* = .71) using a German version of the Buss-Perry Aggression Scale [8] proposed by Amelang and Bartussek [40]. The German version contains the same 29 items and a similar response format to the original version (1 = extremely uncharacteristic of me, 5 = extremely characteristic of me).

#### Income

The participants were asked to specify their annual gross income in Swiss francs. The annual gross income in the sample is above the men's general average income of men in Switzerland, which lies at 86,000 Swiss francs [41].

#### Testosterone

Saliva samples were obtained at 8:00 am using a standardized procedure. Participants were asked to successively fill three salicaps of 2ml capacity (SaliCaps, IBL International GmbH, Hamburg, Germany) with saliva. The saliva samples were stored at −20°C until required for biochemical analysis. Testosterone was analyzed using Luminescence Immunoassay [42] in the biochemical laboratory of the Department of Psychology, University of Zurich. The intra-assay and inter-assay of the Luminescence Immunoassay in saliva are 1.47% and 6.96%, respectively.

### Procedure

Participants independently completed the questionnaires assessing sociodemographic and psychometric data online. Subsequently, they were invited to a single laboratory session at the Department of Psychology of the University of Zurich to provide the biological data. Standardized saliva sampling started at 8:00 am to control for diurnal variation of hormone secretion, and subjects’ awakening time was recorded. Participants were instructed not to consume beverages containing caffeine or alcohol for 48 hours and not to engage in heavy exercise for 24 hours prior to the laboratory session. Three hours before the saliva sampling, they were asked to refrain from smoking, brushing their teeth, chewing gum, and eating. Following the saliva sampling, body weight and height were measured. At the end of the examination, participants were photographed using a digital camera on a tripod while standing upright in front of a white background under standardized lighting. To achieve comparable face photographs, participants were instructed not to smile and to maintain a neutral facial expression while being photographed at a standardized camera distance and angle. The picture with the most frontal and neutral recording of each participant's face was used for the measurement of the fWHR.

### Statistical analyses

Statistical analyses included several steps and were performed using the IBM Statistical Package for the Social Sciences (SPSS Version 22). Statistical significance was defined as *p* < .05. To identify potential confounders, age and body mass index were tested for associations with fWHR by analyzing correlations. There was no significant relationship between age and fWHR (*r* = .03, *p* = .78). As body mass index (BMI) was significantly correlated with fWHR (*r* = .46, *p* = .01), we entered BMI as a control variable in all further analyses. With regard to testosterone, the following potential confounders were tested: age, awakening time, medication intake. Testosterone was not significantly related to age (*r* = .08, *p* = .09) or awakening time (*r* = −.18, *p* = .07). Medication intake as a dichotomous variable was significantly correlated with salivary testosterone (*r* = .20, *p* = .04) and was therefore entered as an additional control variable in the analyses. A one-sample Kolmogorov-Smirnov test revealed that testosterone (*z* = .20) did not deviate from a normal distribution. For testosterone, four cases were missing and were removed from the analyses on testosterone.

First, partial correlations between fWHR and the dependent variables were computed. Second, separate moderation analyses were conducted to examine whether the association between fWHR and the dependent variables was influenced by income. Finally, to test whether testosterone influenced the relationship between fWHR and the dependent variables, further separate moderation analyses were computed. Prior to moderation, all variables were z-standardized and mean-centered. The PROCESS macro [43], an observed variable OLS regression path analysis modeling tool, was used to estimate two-way interactions in the moderation models, as well as simple slopes and regions of significance in order to probe interactions with a moderator. The interactions were probed by testing the conditional effects of fWHR at three levels of the moderator, one standard deviation below the mean, at the mean, and one standard deviation above the mean.

## Results

### Relationship between fWHR, Dark Triad and aggression

Descriptive statistics and intercorrelations among fWHR and the dependent variables are depicted in Table 1. There was no significant correlation either between fWHR and the Dark Triad or between fWHR and aggression.

**Table 1:** Descriptive statistics and intercorrelations among the relevant variables.

	Mean	*SD*	1	2		3		4		5		6		7		8
(1) fWHR	1.99	0.15	1													
(2) Psychopathy	3.40	1.44	0.10	1												
(3) Machiavellianism	2.85	1.39	0.09	0.48	***	1										
(4) Narcissism	4.04	1.71	0.03	0.21	*	0.44	***	1								
(5) Physical Aggression	14.17	3.79	0.01	0.20	*	0.19		0.21	*	1						
(6) Verbal Aggression	13.41	3.28	0.01	0.29	**	0.23	*	0.04		0.24	*	1				
(7) Anger	17.22	5.38	0.02	0.28	**	0.20	*	0.18		0.45	***	0.44	***	1		
(8) Hostility	18.56	5.24	0.04	0.27	**	0.32	**	0.29	**	0.35	***	0.04		0.44	***	1
(9) Testosterone	67.69	26.58	−0.04	−0.06		0.12		0.21		0.11		0.01		−0.03		0.10

*N* = 109. Control variable: BMI. Scale range: Psychopathy (1–9), Machiavellianism (1–9), narcissism (1–9), physical aggression (9–45), verbal aggression (5–25), anger (7–35), hostility (8–40). Testosterone: pg/ml.

SD, standard deviation. Significance levels (two-tailed):

* *p* < .05

** *p* < .01

*** *p* < .001.

### Income as a potential moderator

**Fig 1:**
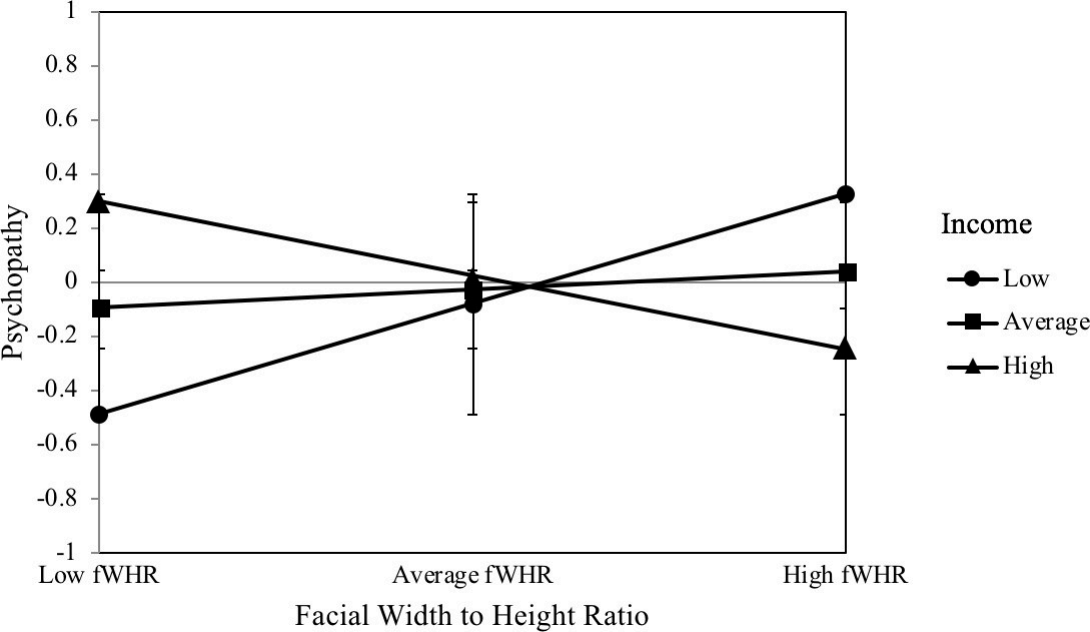
Interaction plot of the relationship between facial width-to-height ratio and psychopathy moderated by income. *N* = 109. Simple slopes of fWHR predicting psychopathy for 1 SD below the mean of income, the mean of income, and 1 SD above the mean of income. Error bars represent the standard deviation.

fWHR was not a significant predictor of psychopathy, *β* = .07, *p* = .59, and the moderator income also did not significantly predict psychopathy, *β* = .05, *p* = .65. In contrast, the interaction between fWHR and income was a significant predictor of psychopathy, *β* = −.34, *p* = .03. The interaction significantly increased the explained variance in psychopathy, *F*(1, 104) = 4.82, *p* = .03, *ΔR*^*2*^ = .05. This interaction is illustrated in Fig 1. fWHR was only a significant predictor of psychopathy at a below-average income, *β* = .41, *p* = .04. fWHR was not a significant predictor of psychopathy at an average income, *β* = .03, *p* = .83, or an above-average income, *β* = −.27, *p* = .19.

**Fig 2:**
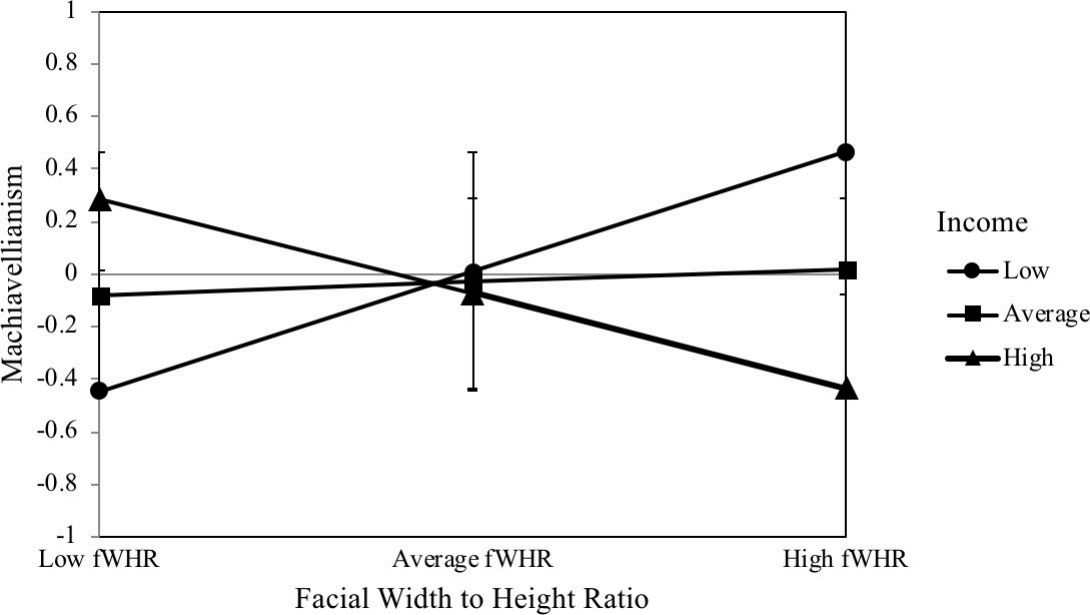
Interaction plot of the relationship between facial width-to-height ratio and Machiavellianism moderated by income. *N* = 109. Simple slopes of fWHR predicting Machiavellianism for 1 SD below the mean of income, the mean of income, and 1 SD above the mean of income. Error bars represent the standard deviation.

fWHR was not a significant predictor of Machiavellianism, *β* = .05, *p* = .65. Similarly, the moderator income did not significantly predict Machiavellianism, *β* = −.04, *p* = .74. In contrast, the interaction between fWHR and income was a significant predictor of Machiavellianism, *β* = −.41, *p* = .01. The interaction significantly increased the explained variance in Machiavellianism, *F*(1, 104) = 7.22, *p* = .01, *ΔR*^*2*^ = .07. This interaction is illustrated in Fig 2. fWHR was a significant predictor of Machiavellianism at a below-average income, *β* = .45, *p* = .01. fWHR was not a significant predictor of Machiavellianism at an average income, *β* = .05, *p* = .65. At an above-average income, fWHR was a marginally significant predictor of Machiavellianism, *β* = −.36, *p* = .07.

fWHR was not a significant predictor of narcissism, *β* = .04, *p* = .71. The moderator income also did not significantly predict narcissism, *β* = .19, *p* = .14. Moreover, the interaction between fWHR and income was not a significant predictor of narcissism, *β* = −.01, *p* = .96.

**Fig 3:**
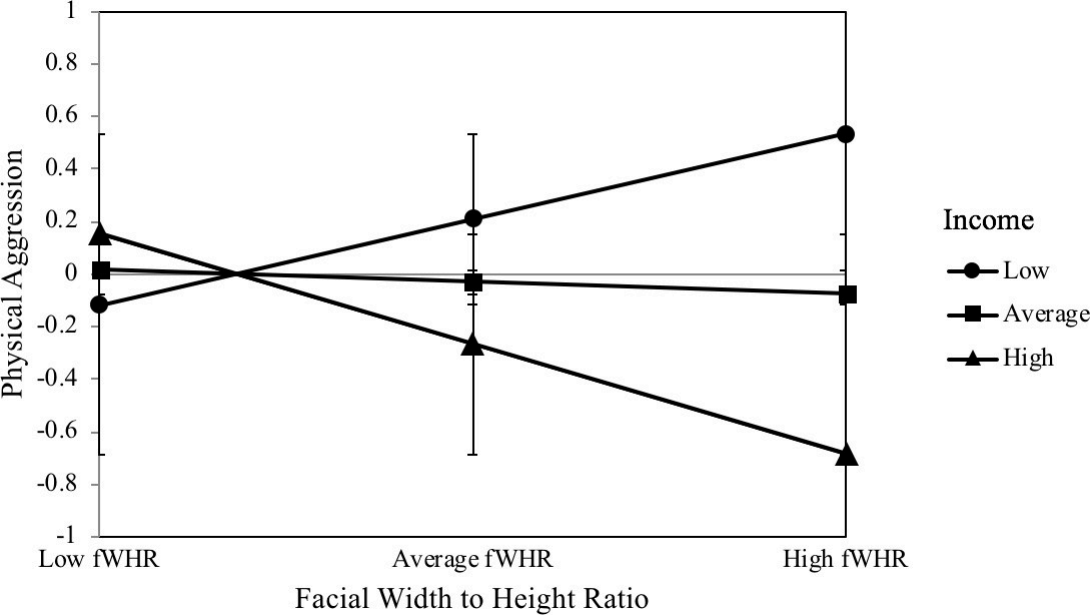
Interaction plot of the relationship between facial width-to-height ratio and physical aggression moderated by income. *N* = 109. Simple slopes of fWHR predicting physical aggression for 1 SD below the mean of income, the mean of income, and 1 SD above the mean of income. Error bars represent the standard deviation.

fWHR was not a significant predictor of physical aggression, *β* = −.05, *p* = .70. The moderator income was a significant predictor of physical aggression, *β* = −.24, *p* = .03. The interaction between fWHR and income was a significant predictor of physical aggression, *β* = −.37, *p* = .03. This interaction significantly increased the explained variance in physical aggression, *F*(1, 104) = 5.09, *p* = .03, *ΔR*^*2*^ = .06; it is illustrated in Fig 3. fWHR was a marginally significant predictor of physical aggression at a below-average income, *β* = .33, *p* = .06, and at an above-average income, *β* = −.42, *p* = .07.

With regard to the other subscales of aggression (verbal, anger, hostility), no significant association was found, either with fWHR or with income. fWHR and income were not significant predictors of verbal aggression, *β* = −.01, *p* = .99 and *β* = .15, *p* = .31, respectively. The interaction between fWHR and income was also not significant, *β* = −.14, *p* = .52. fWHR and income were not significant predictors of anger, *β* = .01, *p* = .96 and *β* = −.15, *p* = .31, respectively. The interaction between fWHR and income was not significant, *β* = −.10, *p* = .60. fWHR and income were not significant predictors of hostility, *β* = .01, *p* = .97 and *β* = −.19, *p* = .24, respectively. The interaction between fWHR and income was also not significant, *β* = −.29, *p* = .15

### Testosterone as a potential moderator

fWHR was not a significant predictor of psychopathy, *β* = .07, *p* = .58. The moderator testosterone was not a significant predictor of psychopathy, *β* = −.05, *p* = .59. Similarly, the interaction between fWHR and testosterone was not a significant predictor of psychopathy, *β* = .03, *p* = .76.

fWHR was not a significant predictor of Machiavellianism, *β* = .09, *p* = .47. The moderator testosterone was a marginally significant predictor of Machiavellianism, *β* = .18, *p* = .06. The interaction between fWHR and testosterone was a marginally significant predictor of Machiavellianism, *β* = .15, *p* = .09. The interaction did not significantly increase the explained variance in Machiavellianism, *F*(1, 99) = 3.02, *p* = .09, *ΔR*^*2*^ = .02.

fWHR was not a significant predictor of narcissism, *β* = .05, *p* = .68. The moderator testosterone was a significant predictor of narcissism, *β* = .27, *p* = .02. Likewise, the interaction between fWHR and testosterone was a significant predictor of narcissism, *β* = .16, *p* = .05. The interaction significantly increased the explained variance in narcissism, *F*(1, 99) = 4.03, *p* = .05, *ΔR*^*2*^ = .02. However, none of the conditional effects of fWHR on narcissism at the three values of testosterone were significant (below average: *β* = −.11, *p* = .47, average: *β* = .05, *p* = .68, above average: *β* = .20, *p* = .12).

With regard to aggression, testosterone was not a significant predictor of physical aggression (*β* = .09, *p* = .38), verbal aggression (*β* = .02, *p* = .90), anger (*β* = −.06, *p* = .59), or hostility (*β* = .08, *p* = .47). Likewise, the interaction between fWHR and testosterone was not a significant predictor of physical aggression (*β* = −.02, *p* = .78), verbal aggression (*β* = −.07, *p* = .96), anger (*β* = −.05, *p* = .68), or hostility (*β* = −.03, *p* = .66).

### Corrections for multiple comparisons

When applying the Holm-Bonferroni method [44], a minimum *p*-value of .0357 would be necessary to render an association significant. All but one of the conducted moderation analyses fulfilled this criterion. The interaction effect of fWHR*testosterone lay above the minimum *p*-value.

## Discussion

### Summary of results

The purpose of this study was to investigate the influences of income, as a key marker of social status, and testosterone on the relationship between fWHR and dominance-related traits. The results show that fWHR is positively linked to self-reports of psychopathy, Machiavellianism, and physical aggression, but only at a below-average income. Furthermore, our findings indicate that fWHR is negatively associated with self-reports of Machiavellianism and physical aggression at an above-average income. Therefore, income was found to be a significant moderator of the relationship between fWHR and physical aggression and of the relationship between fWHR and the two Dark Triad traits psychopathy and Machiavellianism. However, income was not a significant moderator of the relationship between fWHR and the other subtypes of aggression (verbal, anger, hostility). Likewise, income did not moderate the relationship between fWHR and the Dark Triad trait narcissism. In contrast to the other Dark Triad traits, narcissism was positively linked to baseline salivary testosterone levels. Furthermore, testosterone was a significant moderator of the relationship between fWHR and narcissism.

### Interpretation of results

The moderating role of income in the relationship between fWHR and physical aggression corresponds to the findings of a previous study [14], thus demonstrating that fWHR is positively associated with physical aggression, but only in the context of a low income. Furthermore, the results showed that fWHR is positively linked to the two Dark Triad personality traits psychopathy and Machiavellianism, but only in the context of a below-average income. Rather surprisingly, fWHR was negatively linked to Machiavellianism and physical aggression in the context of a high income. The moderating role of income in the relationship between fWHR and the dominance-related traits can be explained by the risk sensitivity theory [29] which predicts that decision-makers prefer high-risk options in situations of high need when lower-risk options are unlikely to meet those needs [45]. Previous research has indicated that fWHR is part of an evolved cueing system of social dominance, aggression, and power [20]. Based on this assumption, men with high fWHRs should display a greater need for power, and consequently perform well in high-status positions [20]. This is also evident in our data, as high-income men with high fWHRs were less Machiavellian and physically aggressive. Accordingly, men with high fWHRs seem to display a better social functioning in high income positions, which can be interpreted in the framework of the risk sensitivity theory. The high social status satisfies the high needs for power and social dominance so that high-risk options are no longer needed. However, when social status is low, men with high fWHRs should be more likely to engage in behaviors linked to impulsivity and social dominance and in risk-taking behaviors [29]. In particular, a low income can represent a condition of high need which motivates the preference for risk-seeking strategies [46] [47]. This may be because low-income men with high fWHRs perceive a discrepancy between their high need for power and the currently low resources to fulfill their needs. To fill this gap, they are more likely engage in risk-taking strategies such as physical aggression, social manipulation (Machiavellianism), or exploitation (psychopathy) in order to (re-)gain control over highly valued resources such as money. In this case of high need, risk-taking serves as a fast and evolutionary strategy to gain significant immediate and evolutionary benefits. Especially under economically harsh and unstable conditions, such as low income, this risk-taking strategy facilitates the opportunistic and strategic exploitation of one's environment to increase reproductive fitness [21]. This would be especially true for low-income men with relatively high fWHRs, who may be more likely to emerge victorious in direct physical altercations [48]. Moreover, risk-taking can constitute an effort to close the gap between low income and high fWHR.

However, fWHR was not linked to the other three subtypes of aggression, namely verbal aggression, anger, and hostility, independently of income. This confirms the findings of a previous study which used the same aggression measure [12]. The most recent study to investigate fWHR and the same aggression measure demonstrated that men's fWHR was only significantly associated with the subscale anger [9]. It is important to note that none of these studies took into account relevant influencing factors such as income, which might explain the inconsistent findings. The present findings showed that fWHR is associated with physical aggression at a relatively low income only. In comparison to the other aggression subtypes, physical aggression is a direct and the most sexually dimorphic aggressive strategy [49]. Since men with high fWHRs have a higher physical formidability and fighting ability [48], they have a greater tendency to apply physical aggression as a risk-taking strategy. This strategy is more advantageous for them because they are more likely to emerge victorious in direct physical altercations.

Previous studies assumed testosterone to be the potential underlying mechanism of links between fWHR and dominance-related traits [30, 31]. The present findings showed that fWHR was not associated with baseline testosterone. This contradicts the findings of Lefevre et al. [30], who demonstrated a small positive correlation between fWHR and baseline testosterone. However, a recent analysis across seven diverse samples of men demonstrated no significant positive relationship between fWHR and baseline testosterone [35]. The authors recommended the use of BMI as a control variable in future studies. In the present data, BMI was strongly correlated with fWHR (*r* = .46) and was therefore entered into the analyses as a control variable. The non-significant results of the present study and of the analysis by Bird et al. [35] suggest that fWHR does not reliably map onto testosterone levels in adulthood. fWHR-neuroendocrine links in men seem to be highly heterogeneous and are therefore apparently not the sole explanatory mechanism for the link between fWHR and dominance-related traits in men. It is plausible that fWHR is more closely tied to exposure to testosterone in adolescence: The link between fWHR and dominance-related behaviors and traits might emerge due to the common influence of testosterone on the craniofacial growth and the expression of behaviors and traits as part of sexual differentiation in puberty [7]. Indeed, administration of testosterone to males with delayed puberty affects various indices of craniofacial growth [50]. Moreover, Welker and colleagues found a positive association between fWHR and pubertal testosterone [33], and pubertal testosterone can bring about long-lasting effects on behavior and personality [51]. These findings provide indirect support for the assumption that fWHR and associated dominance-related traits may be more closely tied to pubertal testosterone exposure than to circulating concentrations in adulthood.

Aggression was also unrelated to baseline testosterone. With regard to previous research, meta-analytic data showed a positive but weak association (*r* = .08) between testosterone levels and aggression [52]. However, this meta-analysis included only three studies investigating the relationship between testosterone and aggression in men older than 35 years. With the exception of the Massachusetts Male Aging Study, with *N* = 1679 participants [53], the included studies had relatively small sample sizes (*N* < 16). The correlation coefficient between testosterone and aggression (anger-out) in the Massachusetts Male Aging Study (*r* = .02) is in the same range as that in the present sample (*r* = .01), which supports our result in terms of external validity. The non-significant results in the present study can be explained in two ways. First, the challenge hypothesis [54] states that testosterone levels rise only in direct response to challenges. Lefevre et al. [30] found positive associations between fWHR and testosterone reactivity following exposure to potential mates. Second, the present study assessed physical aggression as a general trait of aggressiveness by self-report. It is plausible that behavioral measures of aggression are more closely linked to testosterone levels in men. High testosterone levels in male prisoners have been linked to having a history of rape, murder, and armed robbery [55]. It is also important to note that testosterone is not a specific biomarker for aggression but rather for social dominance in general [56].

Regarding the Dark Triad, only narcissism was significantly associated with baseline testosterone. This is in line with the only previous study so far to have investigated the Dark Triad traits and testosterone [57]: A correlation analysis between testosterone and narcissism revealed a significant coefficient of *r* = .18, which is similar to the coefficient of *r* = .22 in the present sample. In both studies, no significant relations emerged for psychopathy or Machiavellianism. The present results extend the existing findings by showing that testosterone moderated the relationship between fWHR and narcissism. In contrast, income moderated the relationship between fWHR and the other Dark Triad traits Machiavellianism and psychopathy. Our findings confirm the assumption that the Dark Triad personality traits clearly overlap (see Table 1) but that they differ on a bio-psychosocial basis. Previous research suggested that narcissism is unique, while Machiavellianism and psychopathy are uniform and united as the "Malicious Two" [58, 21, 59]. Narcissism's unique contribution to the Dark Triad may lie in its strong sense of entitlement ("I tend to expect special favors from others", "I tend to want others to pay attention to me") and grandiosity ("I tend to want others to admire me", "I tend to seek prestige or status") [21]. Grandiosity refers to a highly unrealistic sense of superiority—a sustained view of always being better than others—and is rather an overt presentation of grandiose fantasies than a realistic assessment of actual circumstances. In this sense, grandiosity means "the more the better", and consequently, there is never sufficient achievement or material objects (such as money) to match the internal image of grandiosity. Thus, the present findings suggest that the risk-sensitivity theory applies differently for narcissism: Men scoring high on narcissism do not only seek status and prestige at a low income; rather, they have a high need for infinite superiority at all levels of social status. Moreover, the present findings suggest that testosterone is linked to narcissism by regulating the high need for superiority. The results also show that the interaction between fWHR and testosterone is positively associated with narcissism. This simultaneous impact of fWHR and testosterone on narcissism can be explained by their common overlap with superiority and alpha status in men, which fits with the concept of subclinical narcissism.

### Limitations

Due to the cross-sectional design of the study, our results cannot be interpreted in terms of causal directionality. Additionally, as we conducted moderation analyses without correcting for multiple comparisons, the results need to be interpreted with caution. The identified moderation effects might serve as initial starting points to further investigate the potential effects using experimental designs. Furthermore, as aggression and the Dark Triad rely exclusively on self-report measures, they suffer from the same shortcomings that limit all self-report findings (e.g. socially desirable responding). Nonetheless, research has shown that subjective ratings are relevant in predicting outcomes [60, 61]. For instance, the Dark Triad traits represent valid predictors of antisocial behavior [62, 63]. Furthermore, a meta-analysis showed that computerized surveys, as applied in the present study, generate the most truthful responses [64].

### Directions for future research

Future research should combine subjective and behavioral measures to investigate the influence of fWHR on dominance-related behaviors. It will be necessary to assess the extent to which fWHR and its interaction with income map onto other trait and behavioral outcomes in women. Furthermore, as a recent study showed that the effect of the vertical component of fWHR plays a different role during the formation of social impressions compared to the horizontal component [65]; future research may further investigate the single components of the fWHR and their specific relationship with personality traits. It would also be interesting to compare the different effects of objective and subjective social status on the relationship between fWHR and outcomes of social dominance.

### Conclusion

This is the first study to show that income and testosterone have different influences on the relationship between fWHR and self-reports of dominance-related traits. fWHR is associated with physical aggression and the Dark Triad traits psychopathy and Machiavellianism, but only at a low income. In contrast, testosterone influences the relationship between fWHR and the Dark Triad trait narcissism. Although narcissism strongly overlaps with psychopathy and Machiavellianism, it is the only Dark Triad trait to be associated with baseline testosterone. To conclude, the findings of the present study contribute to and extend research on fWHR and dominance-related traits. They highlight the importance of considering social and neuroendocrine factors when examining associations between fWHR and individual differences in complex human behavioral traits. The findings reveal that fWHR constitutes an evolved cue for social dominance, but there are important context and social factors which exert a significant influence on the informative value of facial cues, such as the fWHR. The findings also provide a basis for future research in order to gain a better understanding of antisocial behavior.

## Supporting information

S1 File Complete items of the questionnaires used. (DOCX)
