# Supplementary material for: The influence of income and testosterone on the validity of facial width-to-height ratio as a biomarker for dominance
Source: PLoS One. 2018 Nov 9;13(11):e0207333. doi: 10.1371/journal.pone.0207333 (PMC6226197; doi:10.1371/journal.pone.0207333)
Supplement: S1 File — (DOCX) [file pone.0207333.s001.docx]

**Supporting Information**

1. **Perry-Buss Aggression Scale**
   1. **English**

The original version of the Perry-Buss Aggression Scale can be found at page 454 in: Buss & Perry (1992). The aggression questionnaire. *Journal of Personality and Social Psychology, 63*, 452-459.

- 1. **German**

**Bitte kreuzen Sie an, wie zutreffend folgende Aussagen für Sie persönlich sind.**

1. Meine Freunde sagen, ich sei etwas streitlustig.

1. Völlig unzutreffend
2. Eher unzutreffend
3. Weder noch
4. Eher zutreffend
5. Völlig zutreffend

2. Wenn Leute besonders nett zu mir sind, frage ich mich, was sie von mir wollen.

1. Völlig unzutreffend
2. Eher unzutreffend
3. Weder noch
4. Eher zutreffend
5. Völlig zutreffend

3. Wenn es sein muss, verteidige ich meine Rechte auch mit Gewalt.

1. Völlig unzutreffend
2. Eher unzutreffend
3. Weder noch
4. Eher zutreffend
5. Völlig zutreffend

4. Manchmal verzehrt mich Eifersucht.

1. Völlig unzutreffend
2. Eher unzutreffend
3. Weder noch
4. Eher zutreffend
5. Völlig zutreffend

5. Manchmal spielt mir das Leben übel mit.

1. Völlig unzutreffend
2. Eher unzutreffend
3. Weder noch
4. Eher zutreffend
5. Völlig zutreffend

6. Ich werde häufiger in Schlägereien verwickelt als andere.

1. Völlig unzutreffend
2. Eher unzutreffend
3. Weder noch
4. Eher zutreffend
5. Völlig zutreffend

7. Es passiert mir oft, dass ich mit anderen nicht übereinstimme.

1. Völlig unzutreffend
2. Eher unzutreffend
3. Weder noch
4. Eher zutreffend
5. Völlig zutreffend

8. Wenn andere mit mir nicht übereinstimmen, kann ich mich nicht zurückhalten, mit ihnen darüber zu streiten.

1. Völlig unzutreffend
2. Eher unzutreffend
3. Weder noch
4. Eher zutreffend
5. Völlig zutreffend

9. Ich sage es meinen Freunden offen, wenn ich anderer Meinung bin als sie.

1. Völlig unzutreffend
2. Eher unzutreffend
3. Weder noch
4. Eher zutreffend
5. Völlig zutreffend

10. Ich frage mich, warum ich manchmal so verbittert bin.

1. Völlig unzutreffend
2. Eher unzutreffend
3. Weder noch
4. Eher zutreffend
5. Völlig zutreffend

11. Ich rege mich schnell auf, aber mein Ärger verraucht auch schnell wieder.

1. Völlig unzutreffend
2. Eher unzutreffend
3. Weder noch
4. Eher zutreffend
5. Völlig zutreffend

12. Ich kann mir keinen Grund vorstellen, weshalb ich jemals eine andere Person schlagen würde

1. Völlig unzutreffend
2. Eher unzutreffend
3. Weder noch
4. Eher zutreffend
5. Völlig zutreffend

13. Ich weiss, dass meine "Freunde" hinter meinem Rücken über mich sprechen.

1. Völlig unzutreffend
2. Eher unzutreffend
3. Weder noch
4. Eher zutreffend
5. Völlig zutreffend

14. Einige meiner Freunde halten mich für einen Hitzkopf.

1. Völlig unzutreffend
2. Eher unzutreffend
3. Weder noch
4. Eher zutreffend
5. Völlig zutreffend

15. Manche Leute haben mich schon so weit gebracht, dass wir uns geprügelt haben.

1. Völlig unzutreffend
2. Eher unzutreffend
3. Weder noch
4. Eher zutreffend
5. Völlig zutreffend

16. Wenn mich jemand schlägt, schlage ich zurück.

1. Völlig unzutreffend
2. Eher unzutreffend
3. Weder noch
4. Eher zutreffend
5. Völlig zutreffend

17. Wenn mich Leute verdriessen (stark verärgern), sage ich ihnen, was ich über sie denke.

1. Völlig unzutreffend
2. Eher unzutreffend
3. Weder noch
4. Eher zutreffend
5. Völlig zutreffend

18. Es fällt mir schwer, meinen Zorn zu kontrollieren.

1. Völlig unzutreffend
2. Eher unzutreffend
3. Weder noch
4. Eher zutreffend
5. Völlig zutreffend

19. Wenn ich nur entsprechend gereizt werde, kann ich jemanden anderen durchaus schlagen.

1. Völlig unzutreffend
2. Eher unzutreffend
3. Weder noch
4. Eher zutreffend
5. Völlig zutreffend

20. Manchmal fühle ich mich wie ein Pulverfass, jederzeit bereit zu explodieren.

1. Völlig unzutreffend
2. Eher unzutreffend
3. Weder noch
4. Eher zutreffend
5. Völlig zutreffend

21. Manchmal kann ich dem Verlangen, eine andere Person zu schlagen, nicht widerstehen.

1. Völlig unzutreffend
2. Eher unzutreffend
3. Weder noch
4. Eher zutreffend
5. Völlig zutreffend

22. Ich habe schon Leute bedroht, die ich gut kenne.

1. Völlig unzutreffend
2. Eher unzutreffend
3. Weder noch
4. Eher zutreffend
5. Völlig zutreffend

23. Ich brause manchmal wegen Nichtigkeiten auf.

1. Völlig unzutreffend
2. Eher unzutreffend
3. Weder noch
4. Eher zutreffend
5. Völlig zutreffend

24. Ich bin schon so ausgerastet, dass ich Gegenstände zerschlagen habe.

1. Völlig unzutreffend
2. Eher unzutreffend
3. Weder noch
4. Eher zutreffend
5. Völlig zutreffend

25. Gegenüber allzu freundlichen Fremden bin ich misstrauisch.

1. Völlig unzutreffend
2. Eher unzutreffend
3. Weder noch
4. Eher zutreffend
5. Völlig zutreffend

26. Glück scheinen immer nur die anderen zu haben.

1. Völlig unzutreffend
2. Eher unzutreffend
3. Weder noch
4. Eher zutreffend
5. Völlig zutreffend

27. Wenn ich frustriert bin, zeige ich meine Verärgerung.

1. Völlig unzutreffend
2. Eher unzutreffend
3. Weder noch
4. Eher zutreffend
5. Völlig zutreffend

28. Ich bin eine ausgeglichene Person.

1. Völlig unzutreffend
2. Eher unzutreffend
3. Weder noch
4. Eher zutreffend
5. Völlig zutreffend

29. Manchmal habe ich das Gefühl, dass andere hinter meinem Rücken über mich lachen.

1. Völlig unzutreffend
2. Eher unzutreffend
3. Weder noch
4. Eher zutreffend
5. Völlig zutreffend
6. **The Dirty Dozen Scale**
   1. **English**

The original version of the Dirty Dozen Scale can be found at page 423 in: Jonason & Webster (2010). *Psychological Assessment, 22*, 420-432. doi: 10.1037/a0019265

- 1. **German**

1. Ich neige dazu, andere zu manipulieren, um meinen Willen durchzusetzen.

1. 1 = Trifft überhaupt nicht zu
2. 2
3. 3
4. 4
5. 5
6. 6
7. 7
8. 8
9. 9 = Trifft vollkommen zu

2. Ich neige dazu, keine Gewissensbisse zu haben.

1. 1 = Trifft überhaupt nicht zu
2. 2
3. 3
4. 4
5. 5
6. 6
7. 7
8. 8
9. 9 = Trifft vollkommen zu

3. Ich neige dazu, von anderen bewundert werden zu wollen.

1. 1 = Trifft überhaupt nicht zu
2. 2
3. 3
4. 4
5. 5
6. 6
7. 7
8. 8
9. 9 = Trifft vollkommen zu

4. Ich habe getäuscht oder gelogen, um meinen Willen durchzusetzen.

1. 1 = Trifft überhaupt nicht zu
2. 2
3. 3
4. 4
5. 5
6. 6
7. 7
8. 8
9. 9 = Trifft vollkommen zu

5. Ich neige dazu, mich nicht um die Moral meiner Handlungen zu kümmern.

1. 1 = Trifft überhaupt nicht zu
2. 2
3. 3
4. 4
5. 5
6. 6
7. 7
8. 8
9. 9 = Trifft vollkommen zu

6. Ich neige dazu, von anderen beachtet zu werden.

1. 1 = Trifft überhaupt nicht zu
2. 2
3. 3
4. 4
5. 5
6. 6
7. 7
8. 8
9. 9 = Trifft vollkommen zu

7. Ich habe Schmeicheleien genutzt, um meinen Willen durchzusetzen.

1. 1 = Trifft überhaupt nicht zu
2. 2
3. 3
4. 4
5. 5
6. 6
7. 7
8. 8
9. 9 = Trifft vollkommen zu

8. Ich neige dazu, gefühllos oder unsensibel zu sein.

1. 1 = Trifft überhaupt nicht zu
2. 2
3. 3
4. 4
5. 5
6. 6
7. 7
8. 8
9. 9 = Trifft vollkommen zu

9. Ich neige dazu, nach Ansehen oder Status zu streben.

1. 1 = Trifft überhaupt nicht zu
2. 2
3. 3
4. 4
5. 5
6. 6
7. 7
8. 8
9. 9 = Trifft vollkommen zu

10. Ich neige dazu, andere für meine Zwecke auszunutzen.

1. 1 = Trifft überhaupt nicht zu
2. 2
3. 3
4. 4
5. 5
6. 6
7. 7
8. 8
9. 9 = Trifft vollkommen zu

11. Ich neige dazu, zynisch zu sein.

1. 1 = Trifft überhaupt nicht zu
2. 2
3. 3
4. 4
5. 5
6. 6
7. 7
8. 8
9. 9 = Trifft vollkommen zu

12. Ich neige dazu, besondere Gefälligkeiten von anderen zu erwarten.

1. 1 = Trifft überhaupt nicht zu
2. 2
3. 3
4. 4
5. 5
6. 6
7. 7
8. 8
9. 9 = Trifft vollkommen zu
